# Probing the chemical–biological relationship space with the Drug Target Explorer

**DOI:** 10.1186/s13321-018-0297-4

**Published:** 2018-08-20

**Authors:** Robert J. Allaway, Salvatore La Rosa, Justin Guinney, Sara J. C. Gosline

**Affiliations:** 1 Sage Bionetworks, 1100 Fairview Avenue N, Seattle, WA 98109 USA; 2 Children’s Tumor Foundation, New York, NY 10005 USA

**Keywords:** Drug targets, Polypharmacology, Webapp, Phenotypic drug screen, Compound-target network

## Abstract

Modern phenotypic high-throughput screens (HTS) present several challenges including identifying the target(s) that mediate the effect seen in the screen, characterizing ‘hits’ with a polypharmacologic target profile, and contextualizing screen data within the large space of drugs and screening models. To address these challenges, we developed the Drug–Target Explorer. This tool allows users to query molecules within a database of experimentally-derived and curated compound-target interactions to identify structurally similar molecules and their targets. It enables network-based visualizations of the compound-target interaction space, and incorporates comparisons to publicly-available in vitro HTS datasets. Furthermore, users can identify molecules using a query target or set of targets. The Drug Target Explorer is a multifunctional platform for exploring chemical space as it relates to biological targets, and may be useful at several steps along the drug development pipeline including target discovery, structure–activity relationship, and lead compound identification studies.

**Electronic supplementary material:**

The online version of this article (10.1186/s13321-018-0297-4) contains supplementary material, which is available to authorized users.

## Background

**Fig. 1:**
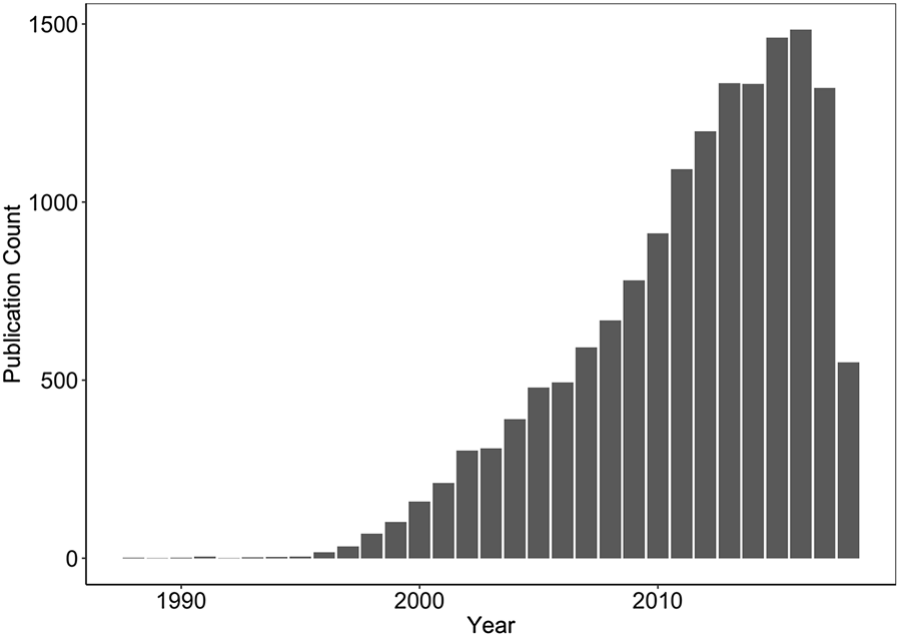
High throughput drug screening is an increasingly common experimental approach. Yearly count of Pubmed-indexed publications that appear with the search term “high throughput drug screening.” Search performed on July 12, 2018

In the modern drug discovery and development process, high-throughput screens (HTS) of drugs have become a common and important step in the identification of novel treatments for disease. In the past decade, studies describing or citing high throughput drug screening are increasingly prevalent, topping 1000 per year for the past 5 years (Fig. 1) and span many disease domains such as cancer, neurodegenerative disease, and cardiopulmonary diseases [1–3]. These screens are often phenotypic in nature whereby a large panel of compounds of known, presumed known, and/or unknown mechanisms of action are tested in a biological model of interest and generate phenotypic readouts such as apoptosis or proliferation. While these types of screens facilitate the rapid identification of biologically active drugs or chemical probes, they also present several challenges.

One prevailing challenge is the identification of the specific biological mechanisms within a cell that determine the response in a screen. The search for novel drugs constantly pushes the pharmaceutical researchers to include novel chemical sets in phenotypic screens, with the caveat that the underlying mechanism of action (MoA) of a particular compound cannot usually be gleaned from the phenotypic screens [4]. Most of the time, identifying the MoA requires additional experimentation, particularly if the molecule represents a novel or understudied chemical entity. Another challenge is that the polypharmacologic nature of many small molecules can make it difficult to interpret HTS results as a given drug may affect multiple targets with a range of efficacy. This, in turn, presents the difficulty of consolidating multiple targets into a unified biological mechanism or set of mechanisms leading to poorly annotated targets, misunderstood MoAs [5], and unknown or ambiguous off-targets with potential deadly side effects [6, 7]. A final challenge is that identification of related molecules and their targets is not always straightforward; in the context of HTS analysis, structurally and functionally related molecules that are not contained in a screening library might be useful to explore.

Multiple tools and databases have attempted to address various aspects of the challenges outlined above (see Table 1). These tools allow the user to explore known polypharmacology of small molecules. Many also allow users to explore compound-target relationships by querying either by molecule or by target: DGIdb, DT-Web, BindingDB, Probes and Drugs, CarlsbadOne, Polypharmacology Browser, STITCH, and SuperTarget allow users to identify MoAs/targets of a given compound by evaluating a query drug [8–15], while DT-Web, BindingDB, Polypharmacology Browser, and STITCH allow users to search by chemical similarity using any query molecule (Table 1). Probe Miner, alternatively, is designed primarily to handle target-based queries [16]. All tools listed in Table 1 allow users to identify molecules with known polypharmacology, but only three, STITCH, SuperTarget, and Probes and Drugs, provide the ability to summarize these targets into biological pathways/mechanisms using a gene list enrichment approach [12, 13, 15]. The final challenge—identifying structurally or functionally related molecules—is addressed by DT-Web, BindingDB, Probes and Drugs, CarlsbadOne, Polypharmacology Browser, and STITCH [9–12, 14, 15].

**Table 1:** Summary of selected features/uses of databases and applications for exploring molecule–target relationships and their overlapping features with the Drug–Target Database

	Drug–Target Explorer	Probe Miner	DGIdb v3.0	DT-Web	BindingDB	Probes and Drugs	Polypharmacology Browser	STITCH	ChEMBLSpace	Carlsbad	SuperTarget
Web app?	X	X	X	X	X	X	X	X		X	X
Open-source software?	X		X	X—underlying R package only	Unknown		X		X		
Search by targets to find drugs?	X	X	X	X	X	X	X—only by PDB-listed ligands	X	X	X	X
Search by drugs to find targets?	X		X	X	X	X	X	X		X	X
Identification of molecules that are associated with multiple query targets?	X			Unknown		X					
Drug structure input?	X			X	X	X	X	X			
Drug name/ID input?	X		X	X	X	X	X	X		X	X
Visualize drug–target networks?	X			X, with user provided drug–target networks	Unknown			X		X	
Identify chemically similar drugs?	X			X, with user provided drug–target networks	X	Scaffold search	X	X		X	X
Allows queries using molecules not in database?	X			X, with user provided drug–target networks	X	X	X	X			
Target organism?	Human	Human	Human	Human	Human and others	Human and others	Human and others	Human and others	Unknown	Human and others	Human and others
Target space?	3.6 k	2.2 k	6.1 k	3.8 k	> 7 k	> 6 k	4.6 k	9.6 mil	Unknown	3.7 k	> 6 k
Chemical space?	280 k	400 k	10 k	4.4 k	> 642 k	> 43 k	870 k	500 k	Unknown	> 435 k	> 196 k
Quantitative interactions?	X	X		Unknown	X	X	X	X	X	X	X
Qualitative interaction?	X	X	X	X		X		Unknown			X
Explore polypharmacology?	X	X	X	X	X	X	X	X	X	X	X
Polypharmacologic target enrichment?	X					X		X			X
Comparison of query molecule to HTS drug response datasets?	X										
Target prediction?				X	X		X				
Database access?	Open	Open	Open	Open	Open	Open	Open	Full database requires license	Unknown	Open	Unknown
Last known update	2018	2018	2018	2018	2018	2018	2016	2016	2015	2014	2012

Related tools include Probe Miner [16], DGIdb [8], DT-Web [9], BindingDB [10], Probes and Drugs [15], CarlsbadOne [14], Polypharmacology Browser [11], STITCH [12], ChEMBLSpace [17], and SuperTarget [13]

While several of the tools listed address one or more of these challenges, there are some gaps (Table 1). For example, ChEMBLSpace does not have a web interface and therefore requires installation on a compatible system before use [17]. In addition, not all of these tools are open-source (STITCH, SuperTarget, BindingDB, Probes and Drugs, CarlsbadOne). An easy to modify open-source application could enable users to create features that are helpful for their specific analyses. While most tools allow both drug-based and target-based queries, none appear to facilitate queries for molecules that affect several targets, which may be useful for users who want to leverage polypharmacology by employing drugs that inhibit multiple biological mechanisms. While multiple targets can be queried at one time in STITCH, it is not straightforward to identify single molecules that affect all query targets. In addition, DGIdb and ChEMBLSpace cannot be used to explore similar chemical space to the query molecule. These two, plus SuperTarget and CarlsbadOne, cannot be queried using molecules that are not in the database; a feature that might help users with novel preclinical candidate drugs. With the exception of DT-Web, STITCH and CarlsbadOne, these tools do not allow visualization of drug–target networks, which may help users address the challenge of identifying structurally or functionally related drugs. No tools other than STITCH and Probes & Drugs perform gene list enrichment, which may help users interpret the biological MoAs of polypharmacologic molecules. Finally, these tools do not allow users to evaluate query drugs in the context of publically available high throughput drug screening datasets.

To address these gaps, we developed the Drug–Target Explorer. Specifically, the Drug–Target Explorer enables the user to [1] look up targets for individual molecules and groups of molecules, [2] explore networks of targets and drugs, [3] perform gene list enrichment of targets to assess target pathways of compounds, [4] compare query molecules to cancer cell line screening datasets, and [5] discover bioactive molecules using a query target and exploration of these networks. We anticipate that the users will include biologists and chemists involved in drug discovery who are interested in performing hypothesis generation of human targets for novel molecules, identifying off-targets for bioactive small molecules of interest, and exploring of the polypharmacologic nature of small molecules.

## Implementation

**Fig. 2:**
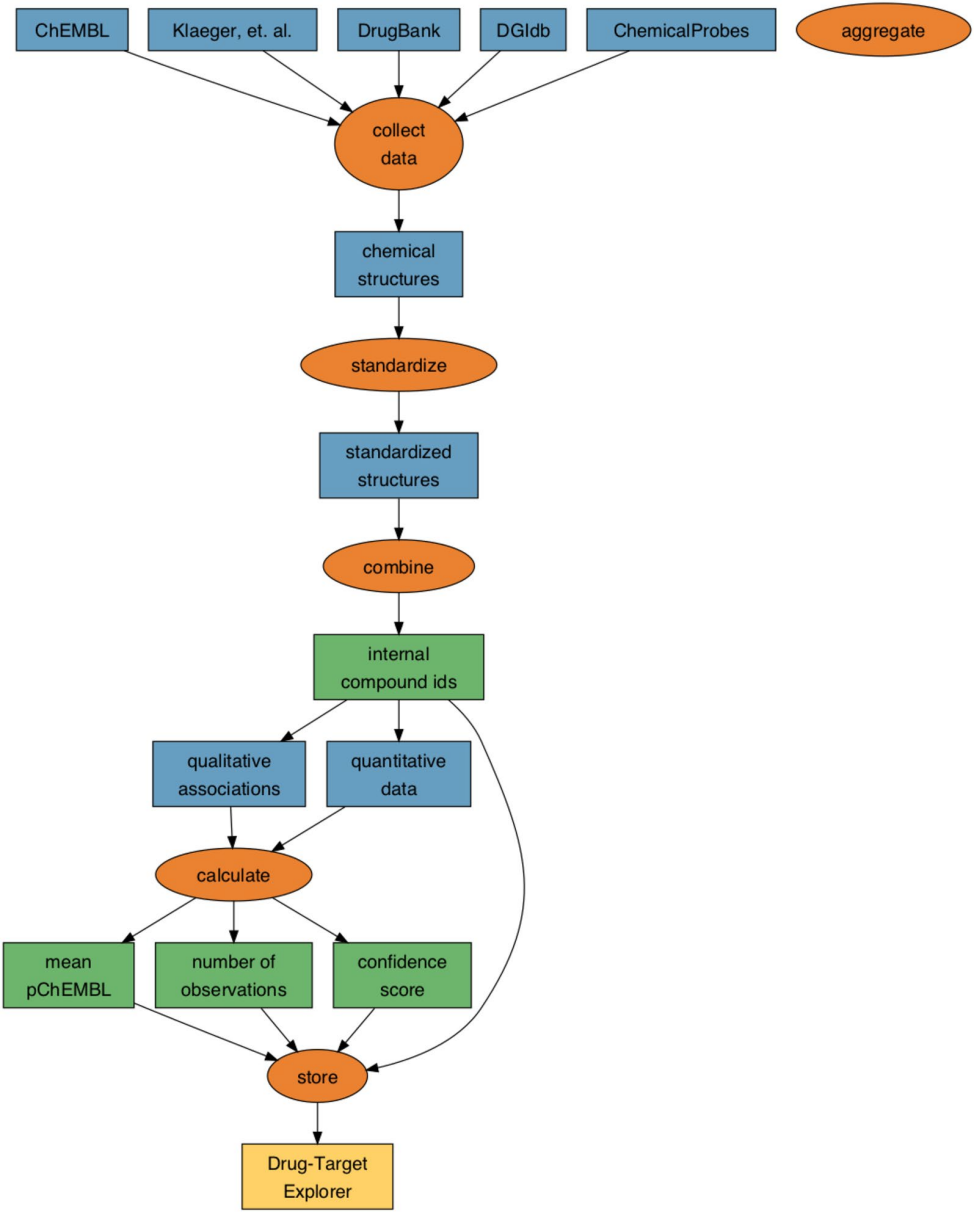
Process for developing the Drug–Target Explorer. Molecule–target and chemical structure data were collected from public sources. In the case of DGIdb, chemical structures were assigned using the PubChem Chemical Identifier Exchange, manually assigned using ChemSpider and PubChem, or mapped to ChEMBL structures by ChEMBL ID. Chemical structures from all source databases were standardized, aggregated, and assigned internal Drug–Target Explorer identifiers. Qualitative and quantitative data were summarized by calculating several summary statistics, and these data were stored together with the internal identifiers to form the Drug–Target Explorer database

To build the database of known compound-target interactions, we aggregated five data sources containing qualitative and quantitative interactions (Fig. 2). We defined a target of a compound as any human protein with a measurable change in activity when directly exposed to that compound, or any human protein qualitatively reported to be directly modulated by that compound in the source datasets. We considered qualitative interactions to be curated compound-target associations with no associated numeric value. These associations are curated and evaluated by experts and are thus a source of high-confidence drug–target information. Quantitative interactions were defined as compound-target information with a numeric value indicating potency of compound-target binding or functional changes. Qualitative compound-target associations were retrieved from the DrugBank 5.1.0 XML database, the DGIdb v3.0.2 interactions.tsv file, and ChemicalProbes.org (acc. July 7 2018) [8, 18, 19]. pChEMBL, IC50, C50, EC50, AC50, Ki, Kd, and potency values for *Homo sapiens* targets were retrieved from the ChEMBL v24.1 MySQL database [20]. Kd values were also obtained from Klaeger et al. 2017, in which the authors determined the Kd of 244 kinase inhibitors against 343 kinases [21]. For all quantitative and qualitative data sources, compound structural information (SMILES) was retrieved when available. When not available, it was batch annotated using the Pubchem Identifier Exchange Service, or, in some cases, manually annotated via PubChem and ChemSpider search [22, 23].

To consolidate data for identical molecules within and across multiple databases, structural strings (SMILES) were standardized using the standardize_smiles function from the MolVS v0.1.1 Python package [24]. Each standardized structure and all external IDs associated with that structure were then assigned an internal identifier, so that groups of molecules with identical structures were assigned to the same internal ID to permit integration of the different datasets. All datasets were combined and summaries were generated for each compound-target comparison using functions from the R ‘tidyverse’ [25]. The number of unique molecules, targets, and molecule–target associations after structural standardization of each source database is described in Table 2.

**Table 2:** Drug–target association metrics obtained from each source database after structural standardization and processing

	ChEMBL v24.1	ChemicalProbes	DGIdb 3.0.2	DrugBank 5.1.0	Klaeger et al.
Molecules	292,765	171	3765	4675	218
Targets	2117	168	1239	2231	348
Associations	650,363	274	8755	11,223	5180

In addition, chemical fingerprints were generated using the R interface (rcdk) to the Java Chemical Development Kit (CDK) [26–28]. The package was modified to use the latest version of the CDK (2.1.1), which enables perception of chiral centers, enabling differentiation between isomeric molecules in circular fingerprints. Circular (functional connectivity fingerprint (FCFP6)-like), MACCS, and extended fingerprints were generated.

The summary metrics described in Table 3 were calculated. One of these metrics, pChEMBL, is used to convey the potency of a given molecule. It is calculated from one of several semi-comparable values in the ChEMBL database, and is defined as the negative log 10 molar of the IC50, XC50, EC50, AC50, Ki, Kd, or potency [20]. Therefore, pChEMBL permits a rough comparison of these values. For example, a pChEMBL value of 7 would indicate that there is a measurable effect on a given target in the presence of 100 nM of molecule. To harmonize the data from Klaeger et al. with ChEMBL data, the Kd values were converted to pChEMBLs. The mean pChEMBL was calculated for every molecule–target combination, as well as the number of quantitative and qualitative associations found in the source databases.

**Table 3:** Drug–target association metrics summarized in the Drug–Target Explorer database

Metric	Unit	Meaning
Mean IC50/AC50/EC50/C50/Potency/Ki/Kd	nM	Mean of values obtained from quantitative datasets; available in database but not app
Mean pChEMBL	− log10 (nM)	Mean − log10 (nM) of all semi-comparable quantitative values
n_qualitative	Count	Number of qualitative associations identified
n_quantitative	Count	Number of quantitative associations identified
Known selectivity index	N/A	The mean pChEMBL for a drug–target association divided by the sum of all pChEMBLs for all drug–target associations for a given drug
Confidence score	N/A	The z-score of the number of quantitative and qualitative interactions found for a drug–target association

We devised a known selectivity index (KSI) for each molecule–target interaction:

$$KSI_{dt} = \frac{{pChEMBL_{dt} }}{{\sum\nolimits_{t \in T} {pChEMBL_{dt} } }}$$

where KSI_dt_ is the known selectivity index for a given molecule–target association, pChEMBL_dt_ is the mean pChEMBL for a specific molecule–target association between drug *d* and target *t*, and ΣpChEMBL_dt_ is the sum of all target (*T*) pChEMBLs for drug *d*.

To quantify confidence in each of the interactions, we calculated a confidence score (c):

$$c_{ab} = \frac{{\left( {n_{ab} + l_{ab} } \right) - \mu_{all} }}{{\sigma_{all} }}$$

where c_ab_ is the confidence of an interaction between molecule a and target b, n_ab_ is the number of quantitative measurements for an interaction between molecule a and target b, l_ab_ is the number of qualitative associations found between molecule a and target b, µ_ab_ is the mean number of associations across all molecule–target associations, and σ_all_ is the standard deviation of all molecule–target associations; subtracting the mean and dividing by the standard deviation converts the confidence score to a z-score. A larger confidence score indicates greater confidence in the relationship between molecule a and target b.

This resulted in a database containing 3650 human targets (represented by HUGO gene symbols), 304,790 small molecules, and 507,059 molecule–target associations summarized from 673,439 quantitative interactions and 18,918 qualitative interactions. Finally, this database as well as fingerprints and chemical aliases for each molecule were saved as R binary files and stored on Synapse. All of the data, as well as snapshots of the source databases used to build the Drug Target Explorer database (with the exception of DrugBank, which requires a license to access) are accessible at www.synapse.org/dtexplorer. The Drug–Target Database is licensed under CC BY-SA 4.0.

**Fig. 3:**
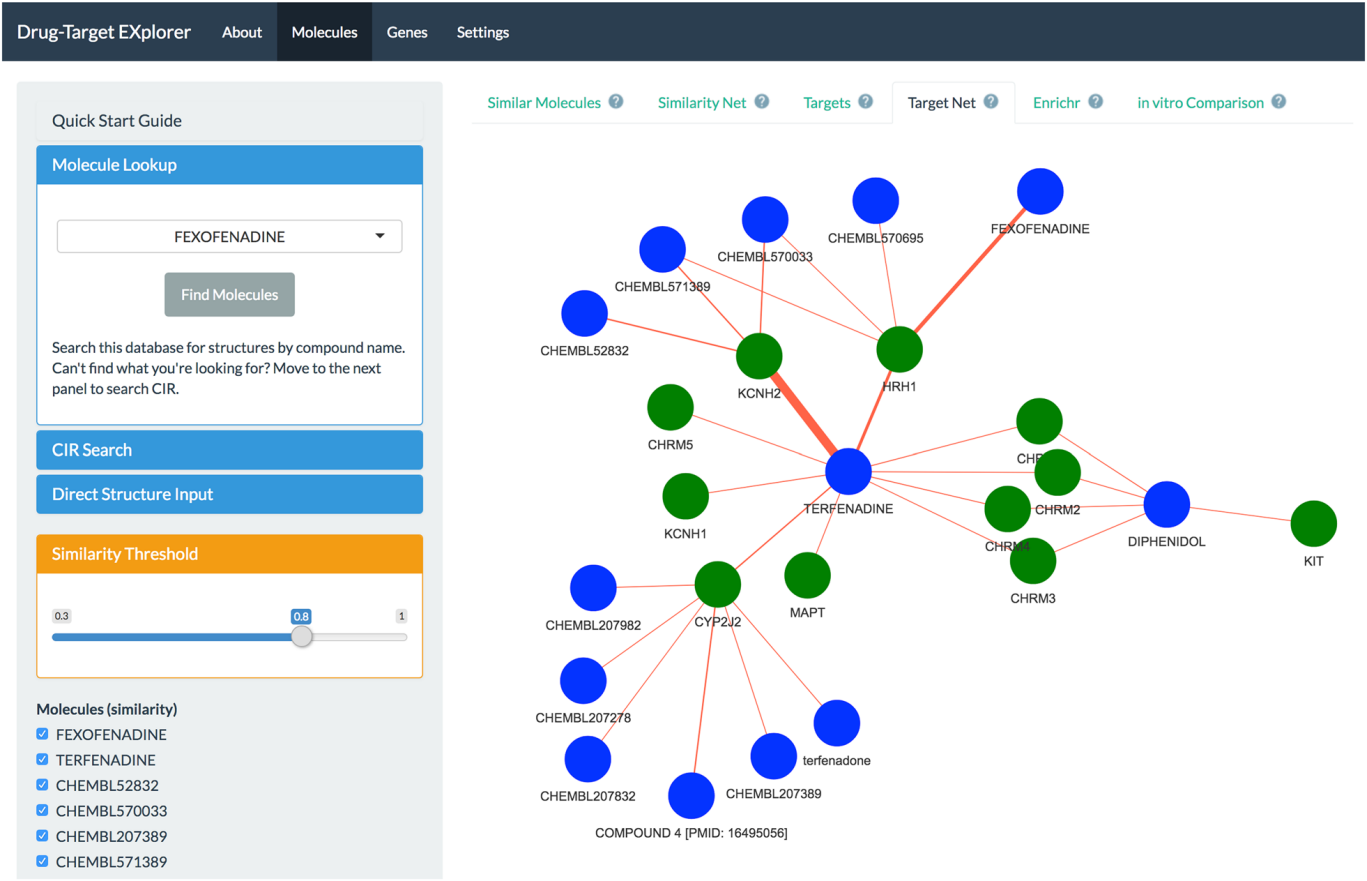
Layout of the Drug–Target Explorer. The “About” tab describes the apps functions and uses, the “Molecules” tab permits molecule-based searching, the “Genes” tab permits target queries, and the “Settings” tab allows the user to pick the fingerprinting method used

We developed a Shiny application to permit exploration of the database [29, 30]. For chemical queries, users can search for molecules in the database by one of three methods: from a list of aliases obtained from the source databases, retrieving the chemical structure using the ‘webchem’ interface to the Chemical Identifier Resolver, or by directly inputting the SMILES string [31]. A Tanimoto similarity threshold allows the user to narrow or widen the chemical space of the results. After querying, the input molecule is converted to a fingerprint and it’s similarity calculated relative to all molecules in the database, using ‘extended’ fingerprints. The user then can view the resulting set of molecules as well as the molecule–target relationships in interactive tables and graphs (Fig. 3). In addition, the user can remove or include molecules on an a-la-carte basis, view the 2D structural representation of the input molecule, and perform target list enrichment analysis [32, 33]. Furthermore, the query molecule can be compared against molecules in the CTRP and Sanger cancer cell line drug-screening datasets to identify identical or similar structures in these datasets, and compare the relationship between chemical structure and correlations in drug response.

For target queries, users can input one or more query HUGO gene(s) and identify molecules that are reported to bind those targets, and view these data in an interactive table. Users can also view these drugs in an interactive graph format to view their association with the query target and their other targets. The Drug–Target Explorer is available at www.synapse.org/dtexplorer. The source code for the Drug–Target Explorer app is available at https://github.com/Sage-Bionetworks/polypharmacology-db. The source code is licensed under Apache 2.0.

## Results

The Drug Target Explorer was designed to facilitate the following use-cases: hypothesis generation of targets for newly-discovered molecules, identification of off-targets for existing bioactive research molecules, and exploration of polypharmacology, and identification of molecules for known targets. Below, we include vignettes highlighting how the Drug–Target Explorer can facilitate analysis in these areas.

### Hypothesis generation of targets for newly-discovered molecules

**Fig. 4:**
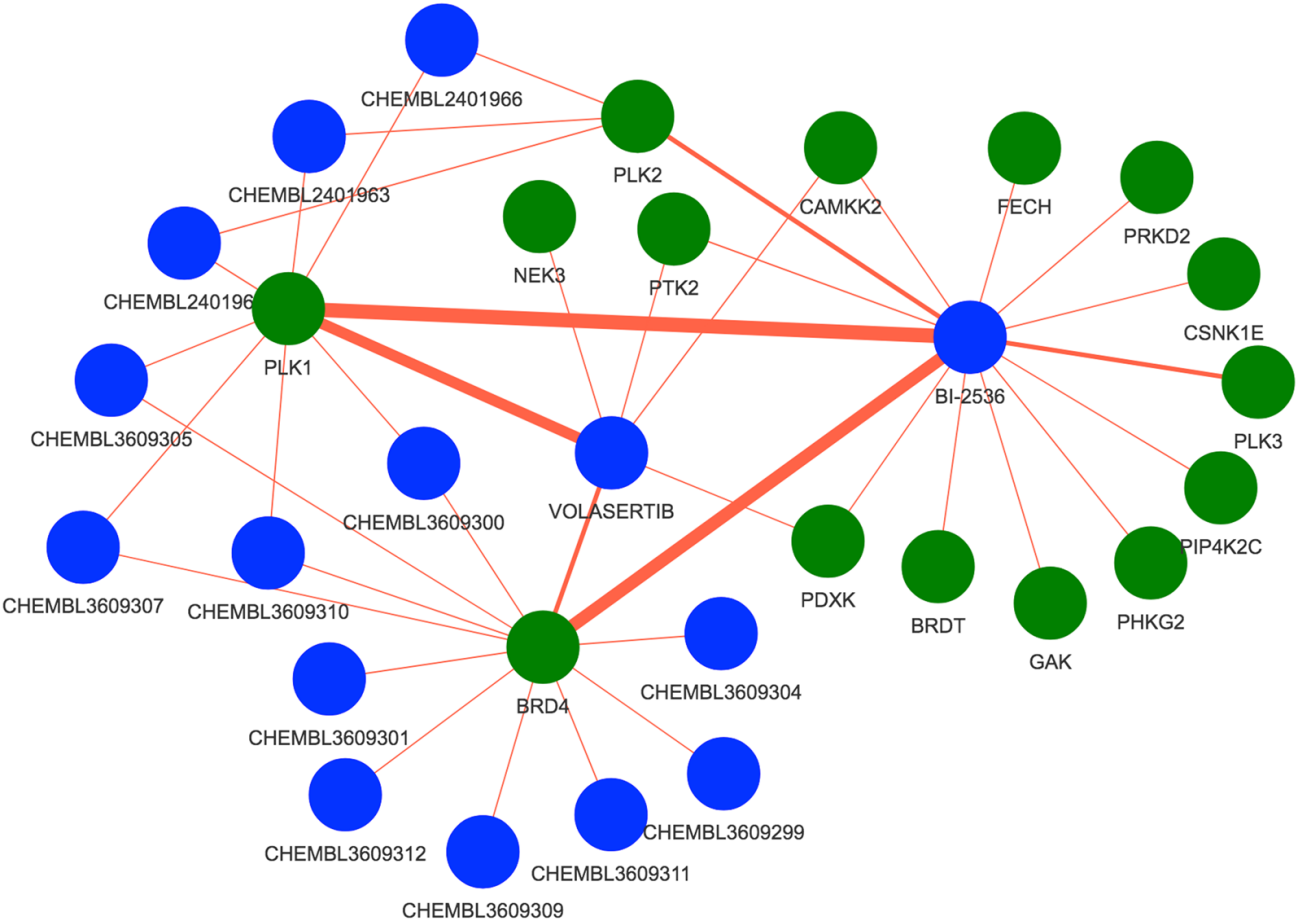
Molecule–target networks highlight targets within chemical families. Using the novel Plk inhibitor C21 as a query with a Tanimoto cutoff of 0.65 (SMILES: CCNC(=O)C1=CC2=C(C=C1)N(C=C2)C1=NC=C2N(C)C(=O)[C@@H](CC)N(C3CCCC3)C2=N1), we identify 15 related molecules (blue vertices), and observe several targets (green vertices) common to multiple members of this group of structurally related molecules, including PLK1, PLK2, BRD4, CAMKK2, PTK2, and PDXK

To highlight the use of this app to find potential off-targets of a novel molecule, we queried the Drug–Target Explorer for C21, a recently-published Polo like kinase (PLK) inhibitor that is not captured in our database [34]. This molecule inhibits Plk2 and Plk1 in the low nM range, and Plk3 in the low uM range [34]. Starting at a similarity of 1, we decreased the similarity cutoff until we identified the most similar molecule in the database at a similarity of 0.74 (CHEMBL3609309), a BRD4-binding molecule. We then continued to decrease the similarity threshold to identify other molecules and targets in the database. At a similarity cutoff of 0.65 we identified 15 molecules (Fig. 4a, Additional file 1: Supplemental Table 1). PLK1 and PLK2 are known targets of several of these molecules, such as BI 2536 and volasertib. Curiously, CAMKK2, BRD4, BRDT, PLK3, PDXK, and PTK2 are also targeted by molecules in this chemical set, with pChEMBL values > 6–8. A plausible hypothesis could be that these targets are affected by this family of molecules, including the query molecule, in the 10–1000 nM range, which would indicate that further research is needed to determine the selectivity of C21 or other structurally related molecules.

### Identification of targets for existing bioactive research molecules

This app may also be useful in identifying alternate targets of existing molecules in a preclinical or exploratory research setting. In order to confidently interrogate the role of cellular targets, one must use compounds with specificity for those targets. A well-known example of a non-specific inhibitor is imatinib. This molecule, developed for use in the treatment of chronic myelogenous leukemia, was initially considered a selective inhibitor of Abl [35]. More recently, several other targets have been identified for imatinib such as KIT, PDGFRA, and PDFGRB [36]. Querying the Drug Target Explorer indicates that there is evidence for 59 targets of imatinib, several of which have pChEMBL values within a reasonable range of Abl, PDGFRA, and PDFGRB (Additional file 2: Supplemental Table 2). These targets must all be considered when evaluating imatinib in human model systems.

A more recent example is the tool compound G-5555, a selective PAK1 inhibitor [37]. This compound has been used to demonstrate the role of PAK1 in cellular processes such as invasion [38]. A search of the Drug–Target Explorer database showed that this molecule not only binds PAK1 (mean pChEMBL = 7.79, Table 4), but there is qualitative evidence for effects on PAK2/3, and quantitative evidence suggesting an effect on SIK2, MAP4K5, and PAK2 at similar concentrations of G-5555 (mean pChEMBLs 8.05, 8, and 7.69 respectively, Table 4). G-5555 also may have an effect on STK family proteins (STK24, STK25, STK26) and LCK. Therefore, any findings with G-5555 with regards to PAK1 inhibition must be validated with other selective inhibitors or genetic approaches, as Jeannott and colleagues did (using other PAK inhibitors such as FRAX597 and FRAX1036, as well as PAK1 silencing RNA), to confirm that the effects observed are PAK1 specific [38].

**Table 4:** Targets of G-5555 found in the Drug–Target Explorer Database

Molecule name	HGNC symbol	Mean pChEMBL	n Quantitative	n Qualitative	KSI	Confidence
CHEMBL3770443	PAK1	7.79	4	1	0.127	2.27
CHEMBL3770443	PAK2	7.69	3	1	0.125	1.64
CHEMBL3770443	LCK	7.28	1		0.119	− 0.229
CHEMBL3770443	MAP4K5	8	1		0.13	− 0.229
CHEMBL3770443	SIK2	8.05	1		0.131	− 0.229
CHEMBL3770443	STK24	7.37	1		0.12	− 0.229
CHEMBL3770443	STK25	7.47	1		0.122	− 0.229
CHEMBL3770443	STK26	7.7	1		0.126	− 0.229
CHEMBL3770443	PAK3			1		− 0.229

### Exploration of polypharmacology and related molecules in biological data

**Fig. 5:**
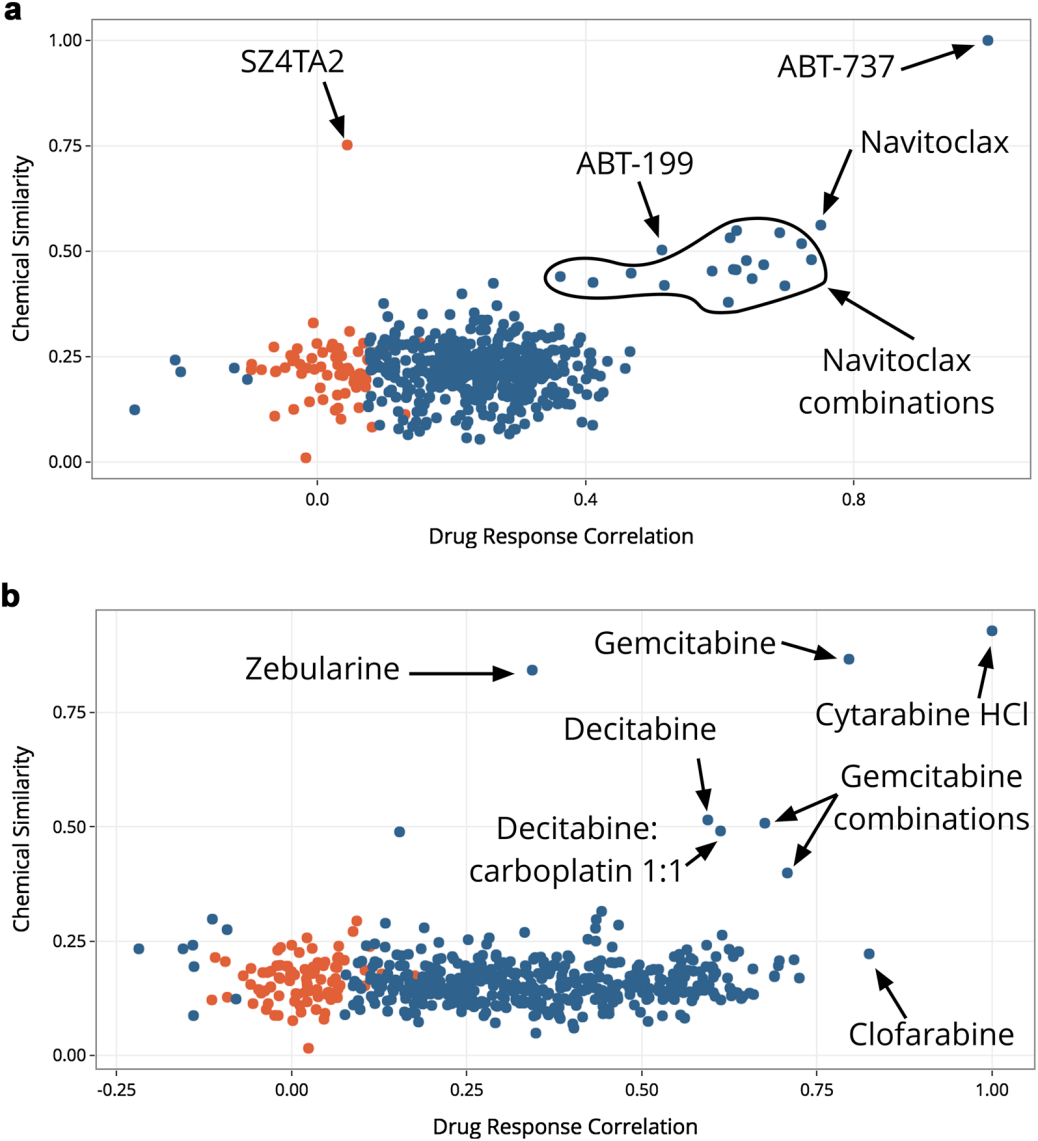
Exploration of similar molecules in biological datasets. **a** A search for ABT-737 identifies similarly-acting BCL family inhibitors in the CCLE dataset, including ABT-199, navitoclax, and combinations of navitoclax with other drugs. **b** A search for 2′-deoxycytidine identifies the most structurally similar molecule in the CCLE dataset (cytarabine HCl) and identifies several molecules with highly and significantly correlated drug responses, including clofarabine, gemcitabine, and decitabine

In order to provide biological context, this app allows the user to aggregate multiple targets from compounds into functional categories. Using the previous example of G-5555, we performed enrichment analysis on the list of targets to identify potential biological pathways that this molecule may disrupt. In doing so, we observed that G-5555 targets are enriched in several Gene Ontology terms and KEGG Pathways like T cell receptor signaling (p = 1.62e-6), Ras/MAPK signaling (p = 3.13e-4), and Golgi-localized proteins (p = 2.86e-2), among several others (Additional file 3: Supplemental Table 3). The app also allows the user to compare the query molecule to drugs in the Cancer Cell Line/CTRP and GDSC/Sanger cell line screening datasets. Specifically, the app identifies the most similar molecule (Tanimoto similarity) available in these datasets and uses that molecule as a reference to calculate the Spearman correlation of drug response across all drugs within the dataset. For example, a search for ABT-737, a BCL family inhibitor, (Fig. 5a) tests the Spearman correlation of AUCs in all cell lines treated with ABT-737 to the AUCs across all cell lines for all other drugs in the CCLE dataset. The app plots these correlations with respect to the Tanimoto similarity of each CCLE molecule to the *input* molecule. The plot shows several molecules with highly and significantly correlated biological activity to ABT-737 (such as other BCL family inhibitors, navitoclax, ABT-199, and combinations with navitoclax) that appear to be more chemically similar to ABT-737 than the majority of the CCLE-tested drugs. Interestingly, this also reveals that SZ4TA2, a BCL-X_L_ inhibitor, while structurally related to ABT-737, does not have a correlated biological response in the CCLE dataset.

This approach can also be used to explore the landscape of chemically similar drugs even when the query drug is not in the drug screening datasets. For example, in Fig. 5b, a query for 2′-deoxycytidine, which is not in the CCLE dataset, identifies cytarabine hydrochloride as the most structurally similar molecule in the CCLE screening set. Using this molecule as a reference, the app calculates the drug response Spearman correlation with the other drugs in the dataset and identified several other molecules that are structurally similar to the input molecule. For example, gemcitabine is quite structurally similar to the input molecule (2′-deoxycytidine) (> 0.8) and has a highly correlated drug response to the reference molecule (cytarabine HCl) (> 0.75). We can also find examples of drugs that are structurally distinct but functionally similar (clofarabine) as well as drugs that are functionally distinct but structurally similar (zebularine).

### Identification of molecules for known targets

**Fig. 6:**
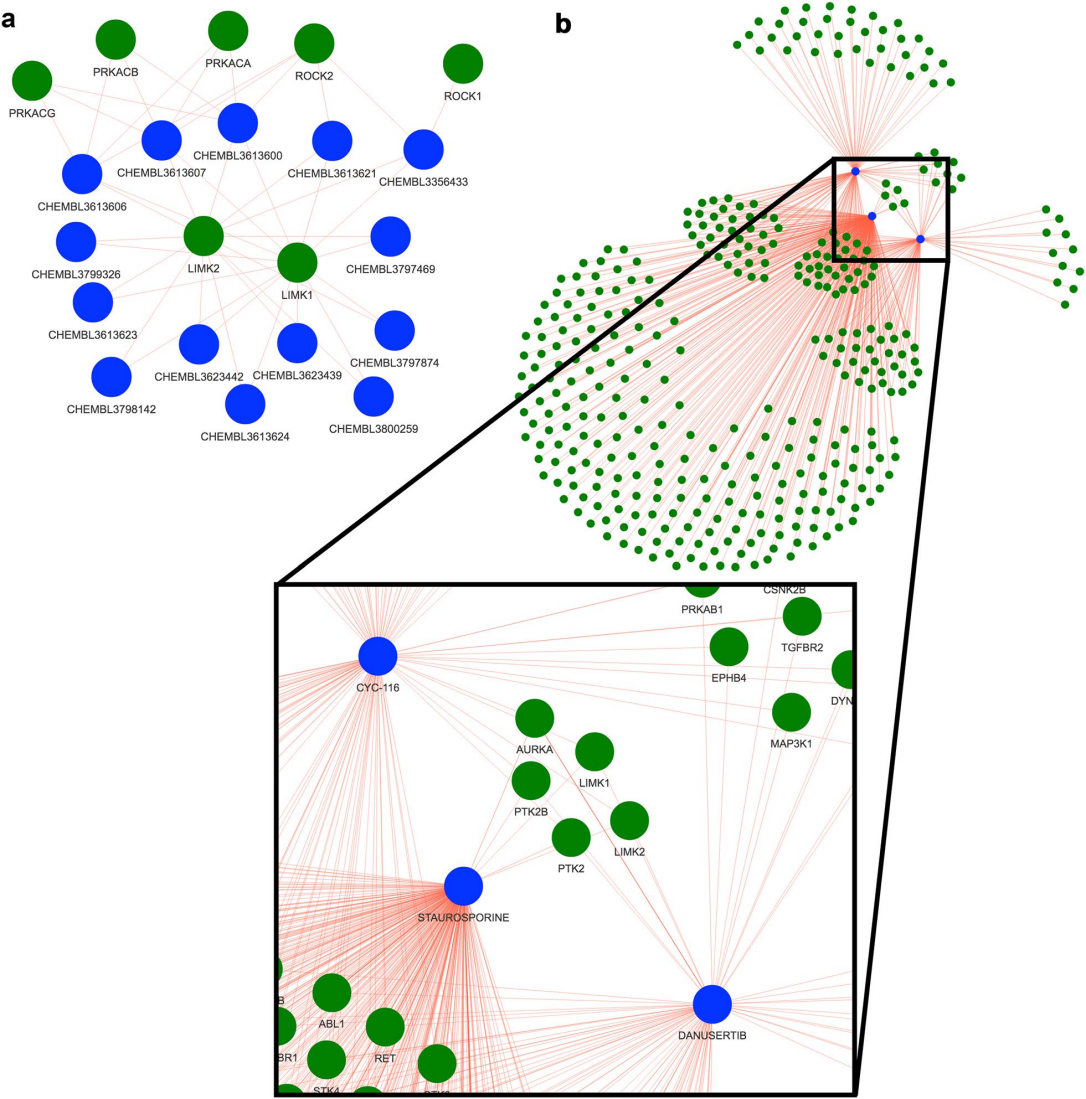
Target-based queries identify molecules that target a gene of interest. **a** A gene-based query for two targets (green vertices), LIMK1 and LIMK2, identifies 10 molecules (blue vertices), as well as other targets affected by these molecules. **b** A query for multiple targets relevant to tumors caused by neurofibromatosis type 2 identifies three molecules that have associations with these targets

Finally, the tool allows users to perform a reverse search, i.e. identify molecules that have an association with a query target or targets and assess the known selectivity of these molecules. For example, Petrilli et al. identified LIM domain kinases as targets of interest in tumors caused by the genetic disease neurofibromatosis type 2 (NF2) [39]. They found that pharmacologic (LIMK1/2 inhibitor BMS-5) and genetic modulation of LIMK1 and LIMK2 caused cell-cycle inhibition and reduced viability in merlin (*Nf2*) deficient Schwann cells [39]. In the context of follow-up studies, it may be beneficial to test alternate molecules that target LIMK1/2 with greater potency than BMS-5. We used the Drug–Target Explorer to find molecules that target LIMK1 and LIMK2 (Additional file 4: Supplemental Table 4, Fig. 6a). For example, BMS-5 (CHEMBL2141887 in the Drug–Target Explorer) has mean pChEMBLs of 7.33 and 7.07 for LIMK1 and LIMK2 respectively, while CHEMBL3623442 has pChEMBLs of 9 and 8.52 for LIMK1 and LIMK2 respectively. Another interesting possibility is the identification of multiple molecules with overlapping desired targets and non-overlapping off-targets to reduce off-target effects, or to identify single-target, multi-drug combinations as outlined by Fitzgerald et al. 2006 [40]. Using the above scenario with LIMK1/2, it may be possible to use structurally distinct molecules in combination or in sequence, like CHEMBL3356433 and others that do not have identical known off-targets (Fig. 6a) to reduce off-target effects while targeting LIMK1/2. The opposite approach could also be taken by finding a single molecule that binds multiple desired targets. In the case of merlin-deficient cells, focal adhesion kinases (FAKs) such as PTK2 (FAK2) and PTK2B, as well as Aurora kinase A (AURKA) have been highlighted as potential targets of interest [39, 41, 42]. Using the Drug–Target Explorer, we can identify molecules that target LIMK1/2, PTK2/2B, and AURKA (Additional file 5: Supplemental Table 5, Fig. 6b). Using this information, a rational hypothesis might be that CYC116 or danusertib could be effective and selective for *NF2*-deficient tumor cells; to our knowledge, the use of these molecules in this setting has yet not been explored.

## Conclusions

In the present study, we demonstrate that the Drug–Target Explorer enables the user to look up targets for novel and known molecules such as C21, G-5555, and imatinib, as well as explore networks of these drugs and their targets. Users can perform target enrichment to consolidate multiple targets to into pathways, compare query molecules to screening datasets, and identify bioactive molecules given a query target.

Several future directions are envisioned for this application. The code and database has been designed in such a way that any database with structural information and drug–gene target information (qualitative associations, or quantitative associations that can be coerced to pChEMBL values) can be harmonized and integrated into the database. Therefore, as new datasets become available, such as the recently-published Drug–Target Commons [43], they can be integrated and released. We also envision occasional errors being identified as the database is explored and vetted by users and have included a feedback form for users to suggest new data to integrate, as well as to highlight necessary corrections to the dataset. Currently, the query molecule to full database similarity calculation is computationally intensive. One solution to speed up calculation times may be to implement a locality sensitive hashing method in future versions of the database and web app, such as the method devised by Cao et al. 2010 [44]. An additional planned feature for this app is the implementation of a bulk annotation feature to allow users to annotate HTS data with targets and/or putative targets of identical or structurally related molecules. Finally, the integration of a predictive framework for identifying targets of query drugs based on drug and target feature data would enable users to quantitatively predict targets of novel molecular entities rather than manually exploring structurally similar molecules.

The Drug–Target Explorer enables users to explore known molecule–human target relationships as they relate to chemical similarity rapidly and with minimal effort. We anticipate that users such as biologists and chemists using chemical probes or studying preclinical therapeutics will find this tool useful in several areas. Specifically, this tool may aid drug discovery efforts by accelerating hypothesis generation, simplifying the transition from phenotypic HTS results to mechanistic studies, and streamlining the identification of candidate molecules that target a protein or mechanism of interest.

### Availability and requirements

- Project name: Drug–Target Explorer
- Project home page: http://www.synapse.org/dtexplorer
- Operating system(s): Platform independent
- Programming language: R
- Other requirements: Chrome, Safari, or Firefox
- License: Apache 2.0

## Additional files

**Additional file 1: Supplemental Table 1.** Targets of C21-like compounds in the Drug–Target Explorer Database. Related to Fig. 2.

**Additional file 2: Supplemental Table 2.** Targets of imatinib in the Drug–Target Explorer Database. Related to Table 2.

**Additional file 3: Supplemental Table 3.** Target enrichment analysis of G-5555 highlights putative mechanistic effects. G-5555 targets were enriched in multiple Gene Ontology terms and KEGG pathways. Related to Fig. 2, Table 2.

**Additional file 4: Supplemental Table 4.** Molecules targeting LIMK1/2. The database was queried for molecules that may modulate LIMK1 and LIMK2; this analysis revealed a large set of putative tool compounds. Related to Fig. 2.

**Additional file 5: Supplemental Table 5.** Identification of multi-kinase-targeting molecules for NF2. A query of the database for molecules that target several kinases of interest in NF2 (AURKA, LIMK1/2, PTK2/2B) identified 3 polypharmacologic compounds. Related to Fig. 2.
